# Detection of Fractured Endodontic Instruments in Periapical Radiographs: A Comparative Study of YOLOv8 and Mask R-CNN

**DOI:** 10.3390/diagnostics15060653

**Published:** 2025-03-07

**Authors:** İrem Çetinkaya, Ekin Deniz Çatmabacak, Emir Öztürk

**Affiliations:** 1 Department of Endodontics, Faculty of Dentistry, Trakya University, Edirne 22030, Turkey; edenizcatmabacak@trakya.edu.tr; 2 Department of Computer Engineering, Faculty of Engineering, Trakya University Edirne 22030, Turkey; emirozturk@trakya.edu.tr

**Keywords:** fractured endodontic instruments, root canal therapy, YOLOv8, Mask R-CNN, Artificial Intelligence, periapical radiography, diagnostic radiology

## Abstract

**Background/Objectives:** Accurate localization of fractured endodontic instruments (FEIs) in periapical radiographs (PAs) remains a significant challenge. This study aimed to evaluate the performance of YOLOv8 and Mask R-CNN in detecting FEIs and root canal treatments (RCTs) and compare their diagnostic capabilities with those of experienced endodontists. **Methods:** A data set of 1050 annotated PAs was used. Mask R-CNN and YOLOv8 models were trained and evaluated for FEI and RCT detection. Metrics including accuracy, intersection over union (IoU), mean average precision at 0.5 IoU (mAP50), and inference time were analyzed. Observer agreement was assessed using inter-class correlation (ICC), and comparisons were made between AI predictions and human annotations. **Results**: YOLOv8 achieved an accuracy of 97.40%, a mAP50 of 98.9%, and an inference time of 14.6 ms, outperforming Mask R-CNN in speed and mAP50. Mask R-CNN demonstrated an accuracy of 98.21%, a mAP50 of 95%, and an inference time of 88.7 ms, excelling in detailed segmentation tasks. Comparative analysis revealed no statistically significant differences in diagnostic performance between the models and experienced endodontists. **Conclusions:** Both YOLOv8 and Mask R-CNN demonstrated high diagnostic accuracy and reliability, comparable to experienced endodontists. YOLOv8’s rapid detection capabilities make it particularly suitable for real-time clinical applications, while Mask R-CNN excels in precise segmentation. This study establishes a strong foundation for integrating AI into dental diagnostics, offering innovative solutions to improve clinical outcomes. Future research should address data diversity and explore multimodal imaging for enhanced diagnostic capabilities.

## 1. Introduction

The identification and accurate localization of fractured endodontic instruments (FEIs) in root canal treatment (RCT) represent a significant diagnostic challenge that directly impacts clinical outcomes. The presence of FEIs obstructs the effective cleaning and disinfection of root canals, thereby compromising the success of endodontic therapy. These challenges point to the critical need for advanced diagnostic techniques to improve precision and reliability in clinical practice [1].

Radiopaque materials aid in detecting FEIs in empty canals but pose challenges in complete RCT, where FEIs and filling materials share similar radiopacity [2]. Comprehensive studies addressing the radiopacity differences between FEIs and root canal fillings, as well as the influence of factors such as anatomical superimposition, geometric distortion, and material-induced artifacts, remain limited in the literature [3]. Advances in digital imaging, including high-resolution sensors and specialized filters, have improved the visibility of small instrument fragments. However, detection remains subjective, time-intensive, and reliant on clinician expertise and image quality [4]. Consequently, alongside advanced diagnostic techniques, the integration of artificial intelligence (AI) models presents a promising approach to improve the accuracy of radiographic examinations and support clinicians in addressing FEI-related challenges [3].

Radiology provides a direct entry point for AI algorithms in dentistry as its digitally encoded images facilitate their application. These algorithms effectively identify variations in tissue density and dental structures, while also detecting and localizing complex features in medical imaging, improving diagnostic accuracy [5].

Recent advancements in convolutional neural networks (CNNs) have shown significant potential in processing large imaging data sets. These algorithms effectively identify variations in tissue density and dental structures, while also detecting and localizing complex features in medical imaging, improving diagnostic accuracy [5].

Among these models, Mask R-CNN and YOLO are notable for their unique capabilities. While previous studies have demonstrated the success of both models in object detection and segmentation tasks [6], these structural differences position Mask R-CNN as more suitable for tasks requiring detailed object-background differentiation, whereas YOLOv8’s rapid detection capability offers a distinct advantage in scenarios demanding quick responses.

Beyond these models, CNNs and long short-term memory networks have recently been employed for the detection of FEIs, achieving notable success. For instance, CNN-based models have shown sensitivity of approximately 81% and specificity up to 87% in identifying FEIs on panoramic images [7]. Similarly, a study employing Mask R-CNN on PAs achieved a mAP exceeding 98%, highlighting its potential for accurate segmentation and localisation of fractured instruments [5]. These methods theoretically enhance diagnostic efficiency by segmenting and localizing fractured instruments. While the diagnostic capabilities of AI models are well-documented, the inclusion of observer comparisons introduces a crucial dimension for assessing their practical applicability in clinical contexts.

The aim of this study is to evaluate the performance of Mask R-CNN and YOLOv8 models in the diagnosis of FEIs and RCTs and to compare their diagnostic capabilities with those of experienced endodontists, thereby assessing their suitability for clinical application.

## 2. Materials and Methods

The research protocol was approved by the Non-interventional Clinical Research Ethical Committee of Trakya University (TÜTF-GOBAEK 2024/384) and was conducted in accordance with the principles of the Helsinki Declaration. Patient names were excluded, and all data were anonymized to ensure confidentiality and ethical compliance.

### 2.1. Data Collection

PAs were selected as the study material due to their accessibility during and after RCT and their common use in cases with complications. Patients from the Department of Endodontics at Trakya University who underwent PA examinations were categorized into the following groups:

(1) FEIs with RCT
(2) FEIs without RCT
(3) Teeth with complete RCT
(4) Teeth without RCT or FEIs

The radiographic images were captured using a Planmeca Pro X™ 2D intraoral X-ray unit (Planmeca^®^, Helsinki, Finland) equipped with a size 2 photostimulable phosphor plate (PSP) detector. Exposure parameters were standardised at 65 kVp, 7 mA, and 0.2 s. Images were digitised using the VistaScan Mini Easy system (Dürr, Biertigheim-Bissingen, Germany), ensuring uniform and reliable imaging quality across all samples.

A total of 396 teeth with FEIs were initially identified, but 16 were excluded for failing to meet quality standards for PAs. Criteria for quality included adequate resolution, absence of distortions, and clear visibility of key anatomical landmarks such as the root apex and canal structure. Radiographs exhibiting artefacts such as cone cuts or motion blurring were excluded. Additionally, 360 teeth with complete RCT and no instrument fractures, as well as 310 teeth without RCT or FEIs, were included. All data were standardised to a resolution of 788 × 612 pixels and stored in PNG format.

### 2.2. Ground Truth Determination and Observer Agreement

**Figure 1:**
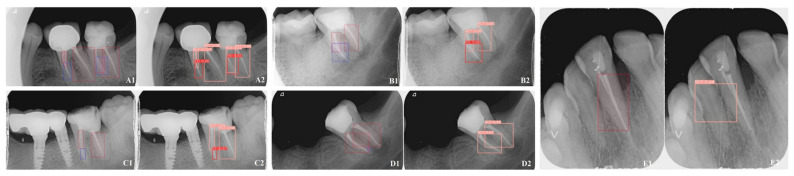
Representative examples of Mask R-CNN’s performance on periapical radiographs (PAs) for detecting fractured endodontic instruments (FEI) and root canal treatments (RCT). The bounding boxes and associated confidence scores highlight the model’s ability to accurately identify and localize objects. Panels (**A1**–**E1**) represent the ground truth annotations marked with blue boxes for FEI and red boxes for RCT, while panels (**A2**–**E2**) depict the segmentations generated by the Mask R-CNN model, where FEI is marked with red boxes and RCT with pink boxes.

Annotations were performed using an open-source tool (CVAT, version 2.2.0). Two endodontic specialists, with 3 and 10 years of experience, respectively, annotated each radiograph for the presence of FEIs and/or RCT using detailed polygonal labelling. Radiographs without these features were left unannotated (Figure 1). To ensure compatibility with analytical models, polygonal annotations were converted to bounding boxes, as Mask R-CNN and YOLO algorithms primarily operate using this format. While Mask R-CNN can generate pixel-level segmentation masks, it first identifies objects using bounding boxes before applying segmentation. YOLO focuses exclusively on bounding boxes for rapid detection [8].

To ensure high inter-observer reliability and accurate bounding box annotations for model training, the Inter-Class Correlation (ICC) analysis was applied with a threshold of 0.9. Radiographs meeting this threshold were considered sufficiently reliable for inclusion in the study. For annotations with ICC values below 0.9, indicating discrepancies between observers, a re-evaluation process was implemented to address these inconsistencies. This comprehensive approach is particularly important in medical imaging studies, where precise localization and accurate annotations are essential for model performance and validity.

In instances where re-evaluation did not resolve disagreements, a third observer was consulted to finalize the annotations. In cases requiring third observer input, the decision-making process was guided by predefined criteria. The third observer assessed 11 radiographs that required further review due to unresolved discrepancies. These disagreements predominantly arose in images where both RCT and FEIs were present, presenting added interpretative complexity.

The final data set included 1050 teeth with corresponding annotations, demonstrating excellent inter-observer agreement and serving as the ground truth for model training. Among these, FEIs were identified in 37.7% (*n* = 396), providing a sample size for robust model evaluation.

### 2.3. Model Selection and Training

Mask R-CNN was chosen for its ability to delineate precise boundaries, making it particularly suited for detecting FEIs within complex anatomical structures. Its advanced segmentation capabilities were advantageous in identifying FEIs within radiopaque areas. YOLOv8 was selected for its rapid detection capabilities, offering practical benefits for clinical decision-making [9,10,11].

**Figure 2:**
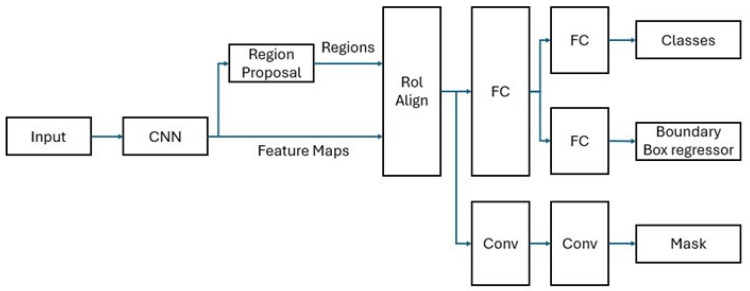
Flowchart of Mask R-CNN architecture. CNN extracts feature maps from the input image. The Region Proposal Network generates candidate regions, which are processed through RoI (Region of Interest) Align to ensure accurate spatial alignment. The extracted features are passed through FC (Fully Connected) layers for classification and bounding box regression. Additionally, Conv (Convolutional) layers are used for mask prediction.

Mask R-CNN employs a two-stage segmentation approach for object detection. In the first stage, the Region Proposal Network (RPN) identifies potential object regions. In the second stage, these regions undergo further analysis: one layer classifies the object, another refines the bounding box, and a third generates the binary mask. To enhance segmentation accuracy, the Region of Interest (RoI) Align layer is utilized [12]. The Mask R-CNN architecture is shown in Figure 2.

**Figure 3:**
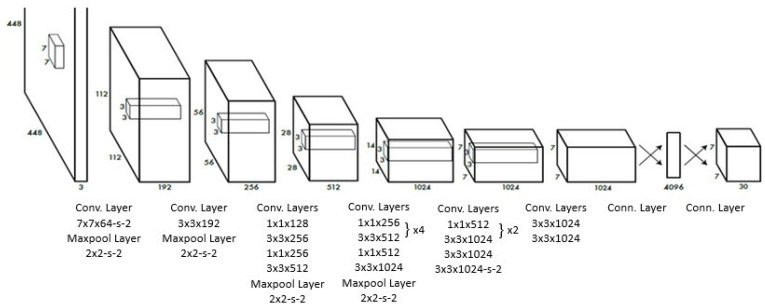
Flowchart of YOLO architecture.

YOLO processes the entire image in a single pass, simultaneously computing bounding boxes and class probabilities using a CNN-based architecture. The CNN output is fed into a prediction stage that determines bounding box coordinates and class probabilities. Due to its single-pass design, YOLO is optimized for real-time applications, prioritizing speed [13]. The YOLO architecture is shown in Figure 3.

The evaluation was performed on a system featuring a Ryzen 7 5700 x processor, 32 GB RAM, and an RTX 4070 Ti GPU, operating on the Windows platform. The data set was divided into training (60%), validation (20%), and test (20%) sets, ensuring class balance across all subsets. Both models were implemented within a structured framework and trained on a curated set of annotated radiographs to ensure accurate detection and localization of FEIs and RCTs. The training process incorporated hyperparameter tuning to optimize performance.

For YOLOv8, key hyperparameters such as the learning rate (initially set at 0.001), batch size (set to 32 samples per batch), and intersection over Union (IoU) threshold (fixed at 0.5) were optimized using a grid search strategy to achieve optimal detection performance. Similarly, for Mask R-CNN, the backbone architecture (ResNet-50), the learning rate schedule (step decay starting at 0.002), and the number of epochs (set to 30) were fine-tuned to enhance segmentation accuracy. Data augmentation techniques were applied to enhance model robustness against variations in radiographic quality and noise. These augmentations help the model generalize better by introducing real-world variations commonly observed in clinical settings, such as horizontal/vertical flipping (50% probability), rotation (± 15° in 10° increments), brightness/contrast adjustments (±20%), Gaussian noise, translation (10% of image size, 10–20% probability), and shear (2°). Rotation ensures diversity in image capture angles without excessive distortion, while translation and shear introduce minor positional shifts reflective of patient or device variability. Flipping leverages dental symmetry to improve feature representation and data set diversity.

### 2.4. Box Loss Calculation

During training, box loss was used to evaluate the distance between predicted bounding boxes and ground truth boxes. L1 and L2 losses were used to minimize localization errors and improve bounding box precision. This metric played a crucial role in refining model accuracy throughout the training process.

### 2.5. Follow-Up Analysis

Six months after the initial annotation, the two observers re-evaluated the data set, and their updated annotations were compared with the predictions of YOLOv8 and Mask R-CNN using confusion matrices. These matrices facilitated a detailed analysis of true positive (TP), true negative (TN), false positive (FP), and false negative (FN) across the classes (FEIs, RCTs, BG), offering insights into observer consistency, model performance, and areas where the models might face challenges. This comparison provided valuable metrics for assessing the effectiveness of the models in detecting and classifying these categories, as well as identifying areas requiring further refinement.

### 2.6. Methods for Evaluating Model Effectiveness

These matrices offered insights into the models’ ability to detect FEIs, RCTs, and background (BG). Key metrics—accuracy, IoU, mAP50, and interference time—are crucial for assessing model performance in detecting and localizing RCTs and FEIs in PAs. Each is detailed below.

Accuracy (Equation (1)) measures the proportion of correct predictions, including both TPs and TNs, out of all predictions made by the model. IoU (Equation (2)), ranging from 0 to 1, was used to measure overlap and assess localization accuracy between predicted and ground truth bounding boxes. mAP50 (Equation (3)) computes average precision at an IoU threshold of 0.50, requiring at least 50% overlap for correct detection. Inference time refers to the time taken by the model to generate a prediction after receiving input. By selecting mAP50 as evaluation metric, we aim to balance accuracy with practical applicability, ensuring our models are both effective and deployable in diverse clinical scenarios.

(1) $$\mathrm{Accuracy}=\frac{TP+TN}{TP+TN+FP+FN}$$

(2) $$\mathrm{IoU}=\frac{Area\;of\;Intersection}{Area\;of\;Union}$$

(3) $$\mathrm{mAP}=\frac{1}{N}\sum_{i=1}^{N}{AP}_{i}$$

### 2.7. Statistical Analysis

A Z-test was conducted to compare accuracy, IoU, and mAP50 between YOLOv8 and Mask R-CNN. Given the sufficiently large sample size (*n* = 1050), the normality assumption was considered valid. The test was based on the null hypothesis (H_0_) that no significant difference exists between the models, while the alternative hypothesis (H_1_) proposed a statistically significant difference. A two-tailed Z-test was applied, with statistical significance set at *p* < 0.05. All statistical analyses were performed using Microsoft Excel (Microsoft 365, Version 2501, 64-bit).

## 3. Results

### 3.1. Model Performance

The evaluation of YOLO v8 and Mask-R-CNN models revealed consistently high performance across key metrics, showcasing their suitability for detection and segmentation tasks (Table 1). Accuracy rates were observed at 97.40% for YOLO v8 and 98.21% for Mask-R-CNN. Despite the slight numerical advantage of Mask-R-CNN, the difference in accuracy between the two models was not statistically significant (*p* = 0.571). Similarly, IoU scores of both models were remarkably high, with YOLO v8 achieving a perfect score of 100% and Mask-R-CNN scoring 99%. This difference also lacked statistical significance (*p* = 0.146), indicating comparable segmentation precision between the two methods.

In contrast, mAP50 metric highlighted a statistically significant difference (*p* = 0.020). YOLO v8 outperformed Mask-R-CNN with a mAP50 of 98.9%, compared to 95% for Mask-R-CNN, suggesting a superior ability to correctly detect and classify objects across varying confidence thresholds.

Another notable distinction was observed in computational efficiency, as measured by interference time. The YOLO v8 model demonstrated a significant advantage in speed, with an interference time of only 14.6 ms, far surpassing the Mask-R-CNN model’s 88.7 ms. This substantial reduction in processing time underscores YOLO v8’s potential for real-time applications, where rapid decision-making is critical.

### 3.2. Algorithmic Overview of YOLOv8 and Mask R-CNN

Explainable AI (XAI) techniques were employed to enhance the interpretability and reliability of object-detection results. Saliency mapping was utilized to identify the most influential regions contributing to the detection of FEIs, providing valuable insights into model transparency and robustness. Based on the documentation [14], the Detector-RISE (D-RISE) saliency algorithm was selected as it aligns with the object-detection approach used in this study. The results were generated accordingly.

In the saliency map, red areas indicate the regions that contribute most to object recognition, while blue areas correspond to regions with minimal relevance. Changes and variations in the red areas are considered to have the highest impact on the decision-making process. In cases where multiple classes are present in the examples, saliency values are provided for a single class. Apart from this, saliency graphs can be calculated separately for each value [15].

**Figure 4:**
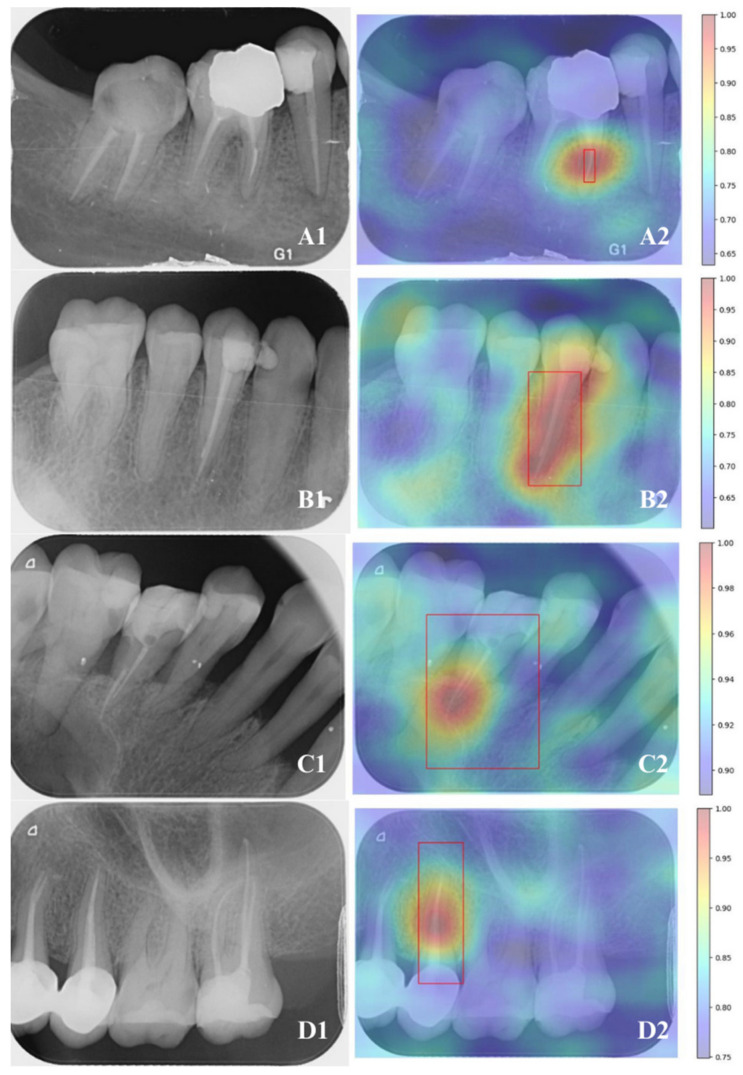
Saliency map outputs for FEI and RCT detection using YOLO and Mask R-CNN. (**A1**–**D1**) Raw periapical radiographs, (**A2**–**D2**) corresponding saliency maps. (**A**) YOLO-based saliency map for FEI detection, (**B**) YOLO-based saliency map for RCT detection, (**C**) Mask R-CNN-based saliency map for FEI detection, and (**D**) Mask R-CNN-based saliency map for RCT detection. The red boxes indicate the regions identified by the models as containing FEI or RCT, highlighting the areas of interest detected by the respective deep learning approaches.

For YOLO and Mask-R-CNN models, saliency map outputs for FEI and RCT examples are presented in Figure 4.

### 3.3. Training and Validation Stability

**Figure 5:**
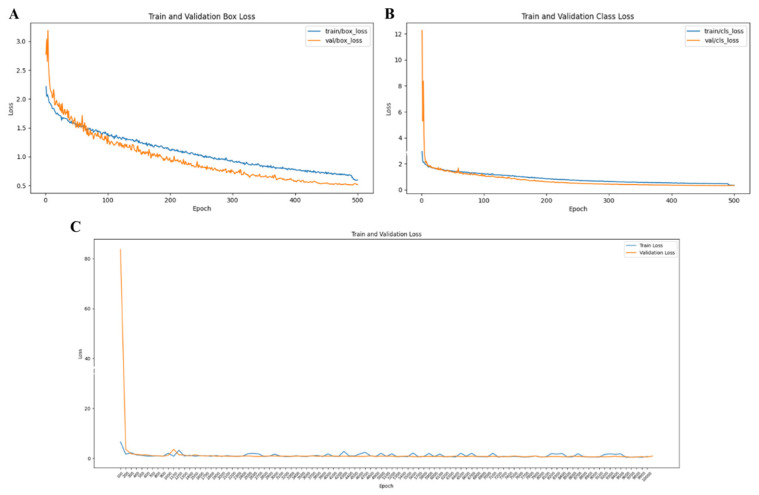
Comparison of training and validation losses for YOLOv8 (top) and Mask R-CNN (bottom) models. The YOLOv8 graphs depict box loss (**A**) and class loss (**B**), illustrating a steady decrease in both training and validation losses with minimal divergence, indicating strong generalization and effective performance in object localization and classification. In contrast, the Mask R-CNN graph (**C**) shows the total loss across training and validation, with training loss decreasing rapidly and validation loss stabilizing with slight fluctuations, reflecting its ability to perform detailed segmentation tasks. Overall, YOLOv8 demonstrates faster convergence and smoother loss reduction, while Mask R-CNN exhibits robustness in tasks requiring precise segmentation.

YOLO v8—Box Loss: Both training and validation box loss steadily decreased, approaching 0.5, indicative of progressive improvements in bounding box predictions. The close alignment between training and validation losses suggests consistent performance across data sets and minimal overfitting (Figure 5A).

YOLO v8—Class Loss: Class loss decreased rapidly during training, reflecting improved accuracy in classifying objects. The alignment of training and validation losses demonstrated the model’s robustness and reduced likelihood of overfitting in classification tasks (Figure 5B).

Mask-R-CNN—Overall Loss: Validation loss peaked early but stabilised rapidly, indicating improved object localisation and classification accuracy. The close alignment of training and validation losses highlighted the model’s strong generalisation capabilities (Figure 5C).

### 3.4. Comparative Performance Between Models and Endodontists

The comparative analysis between the models and endodontists revealed no statistically significant differences, reflecting the consistency and reliability of both human and machine performance (Table 2). For the FEI F1 score, Endodontist A and Endodontist B achieved identical values of 0.9947 (*p* = 1.000). This uniformity extended to comparisons between Endodontist A and the machine learning models, as no significant differences were found for either Mask-R-CNN (*p* = 0.640) or YOLO v8 (*p* = 0.447). Similarly, Endodontist B’s performance did not significantly differ from Mask-R-CNN (*p* = 0.640) or YOLO v8 (*p* = 0.447).

Specifically, the diagnostic accuracy values for Endodontist A and Endodontist B exhibited slight variations over the 6-month period (Table 2). Accuracy values were as follows: 0.9947 at the initial assessment and 0.9947 after 6 months for both endodontists, followed by 0.9944 at both time points. A decrease in diagnostic accuracy was observed in a particular instance, with values dropping to 0.967 for both endodontists. These findings suggest a degree of temporal inconsistency in human diagnostic decisions, focusing on the influence of cognitive and interpretative factors over time.

The RCT F1 score further supported these findings, as no significant differences were detected among the endodontists or between the models (*p* > 0.05). These results suggest that both models perform at a level comparable to experienced practitioners, further validating their applicability in clinical or research settings.

Within-group comparisons of F1 scores also showed no significant variations across metrics. For example, the BG metric did not significantly differ from FEI (*p* = 0.971) or RCT (*p* = 0.064). This lack of statistically significant differences across multiple comparisons indicates a high level of consistency and robustness in both human and model performance.

YOLO v8 and Mask-R-CNN demonstrated performance metrics comparable to skilled endodontists, with added benefits of automation and scalability. YOLO v8 outperformed Mask-R-CNN in computational efficiency and mAP50, making it more suitable for high-speed, accurate applications.

## 4. Discussion

This study demonstrates the accuracy and reliability of YOLOv8 and Mask R-CNN models in detecting and localizing FEIs and RCTs. Both models demonstrated robust performance, with YOLOv8 and Mask R-CNN effectively identifying all FEI cases while maintaining minimal error rates in RCT classification. The reported mAP50 scores for YOLOv8 and Mask R-CNN align closely with results from similar studies [5,16,17], further validating their potential as clinical diagnostic tools.

Accurate identification of FEIs is crucial for effective treatment planning, particularly for less experienced clinicians, as their radiopacity often overlaps with that of RCT materials. A key focus of this study was the challenge of distinguishing between these similarly radiopaque structures, a topic not extensively explored in prior research [3].

Once a FEI is detected, the treatment approach will depend on the affected tooth and canal, the location of the instrument separation, the amount of remaining contaminated material, and the potential damage to the dental structure if removal is attempted [18]. If accessible in the coronal or middle third, retrieval may be attempted using ultrasonic tips or a specialized device [19]. When removal risks excessive dentin loss or perforation, bypassing the fragment allows for successful obturation [20]. In apical third cases where removal is unfeasible, sealing the canal with biocompatible materials minimizes bacterial leakage and preserves endodontic success [21]. AI-assisted detection enhances treatment planning by providing precise localization, aiding clinicians in selecting optimal management strategies [5].

To ensure data set reliability and minimize observer bias, the study employed a strict methodology, exemplified by a high ICC threshold. By introducing an additional layer of complexity, this study required the models to differentiate between multiple scenarios within a single radiograph, including RCT and/or FEIs.

This study utilized YOLOv8 and Mask R-CNN, capitalizing on their respective strengths and differences in dental diagnostics (Table 3). YOLOv8 exhibited exceptional performance in rapid detection and classification, achieving a significantly faster inference time (14.6 ms) and an impressive mAP50 score of 0.989. These attributes may render it particularly well-suited for real-time clinical applications where both efficiency and accuracy are critical. Similarly, Mask R-CNN excelled in precise segmentation tasks, offering detailed visualization of complex dental structures, albeit with a slower inference time (88.7 ms). The slower performance of Mask R-CNN compared to YOLOv8 is not surprising, as Mask R-CNN involves more complex processes, including region proposal generation and pixel-wise segmentation, whereas YOLOv8 is optimized for real-time detection with a single network pass [16,22]. The performance of the models was further assessed by analyzing TP, FP, and FN values for both YOLO and Mask R-CNN, whereas TN values were omitted, as examples without annotations were considered unnecessary (Supplementary Materials, Figures S1 and S2).

A notable finding in this study was the statistically significant difference in mAP50 between the models, emphasizing YOLOv8’s strength in detection accuracy. Future research may benefit from integrating mAP50 with complementary metrics to provide a more comprehensive understanding of model performance across varying scenarios. Additionally, exploring the impact of alternative IoU thresholds could further enhance the adaptability of object-detection systems [23,24].

While mAP50 was the only metric showing a statistically significant difference, this finding should be considered alongside other performance metrics to ensure a holistic evaluation of model capabilities. Different metrics reveal distinct capabilities of models and play equally critical roles in determining their suitability for specific clinical applications. The results underscore the importance of adopting multidimensional evaluation frameworks to fully understand the trade-offs between speed, accuracy, and precision in object-detection models.

Numerous studies highlight the effectiveness of YOLOv8 and Mask R-CNN in detecting and segmenting pathologies in dental radiographs. YOLOv8 has achieved 77.03% accuracy in classifying periodontal diseases in bitewing radiographs and 75% in PAs [25], while our study demonstrated a significantly higher accuracy of 97.4% in detecting FEIs. Similarly, the YOLOv8 m model attained 90% accuracy in diagnosing dental diseases from bitewing and orthopantomographic images, aligning with our findings [22]. Additionally, YOLOv8 reached 95.2% accuracy and 97.5% mAP50 in segmenting mandibular radiolucent lesions, comparable to our results [16]. A study has shown that while CNNs achieve higher classification performance, YOLOv8 exhibits a trade-off between speed and accuracy, with lower F1 scores in detecting apical and peri-endo combined lesions [26]. Additionally, research on the automated detection of dental conditions using YOLOv8 has reported precision and recall values exceeding 80%. However, its performance declines when applied to external data sets, highlighting challenges in generalizability [24].

Mask R-CNN studies underline its reliability in segmentation and anomaly detection. Wang et al. (2024) integrated Mask R-CNN with a neural network classifier for diagnosing periapical diseases, achieving a pixel accuracy exceeding 97% [27]. An attention-enhanced Mask R-CNN achieved 79.5% accuracy [28], similar to the accuracy observed in our FEI detection. Other studies reported 75–80% accuracy in identifying dental caries and periodontitis, highlighting its utility in dental radiographic analysis [29,30]. For FEI detection in PAs, Mask R-CNN achieved 98.8 mAP, 95.2% accuracy, and 97% F1 scores, closely aligning with our findings [5]. Furthermore, it demonstrated 100% accuracy and 97.49 mAP in tooth segmentation and numbering, underscoring its precision and clinical applicability [17].

The comparative analysis between endodontists and AI models revealed comparable levels of diagnostic accuracy, with both achieving high performance metrics. However, a notable finding was the variation observed in the diagnoses made by endodontists after a 6-month interval compared to their initial assessments. These results indicate that while endodontists and AI models demonstrated comparable diagnostic accuracy, temporal variability was evident in endodontists’ assessments over a 6-month period. This fluctuation suggests the influence of cognitive and interpretative factors over time. Such variability may be attributed to memory effects, evolving clinical judgment, or shifts in diagnostic perception. These results highlight the inherent subjectivity in human interpretation and underscore the need for standardized evaluation protocols to enhance diagnostic consistency and reliability in endodontic practice. This highlights the potential for variability in human interpretations over time, likely influenced by memory effects, shifts in judgment, or changes in clinical interpretation.

Specifically, while the overall F1 scores of the endodontists remained high, minor discrepancies were identified in certain cases, particularly in the interpretation of FEIs. This variability emphasizes the inherent subjectivity and temporal variability of human diagnoses, even among experienced practitioners.

In contrast, YOLOv8 and Mask R-CNN demonstrated consistent diagnostic performance over the same data set, unaffected by temporal or cognitive factors. This consistency highlights one of the key advantages of AI systems: their ability to provide stable and reproducible diagnostic outcomes, free from the influence of human-related factors such as fatigue or shifting perceptions.

These findings suggest that a combined approach leveraging both AI models and human expertise could enhance diagnostic accuracy. AI models can serve as reliable and consistent second opinions, helping to mitigate variability in human diagnoses and ensuring a more robust diagnostic process over time.

Despite the high success rate, certain limitations must be acknowledged. The relatively low prevalence of FEIs (37.7%) constrains the data set size, potentially limiting the models’ robustness in real-world applications. However, this limitation reflects the fact that FEIs are relatively rare in clinical settings, which inherently affects data set composition [1]. Additionally, noise and artifacts in PAs, such as anatomical superimpositions and material distortions, present challenges for accurate detection and localization. These limitations emphasize the need for enhanced data quality and more robust training strategies to improve model performance under diverse clinical conditions.

AI, shaped by training data, can reflect inherent biases, necessitating human oversight to ensure fairness. While transparency fosters trust, it also poses privacy risks, highlighting the need for a balance between openness and data protection [31,32]. A significant challenge lies in bridging the gap between AI research and clinical application, as most studies remain experimental. The absence of standardized data protocols and inconsistent labeling further exacerbate biases, limiting AI’s reliability in clinical settings [33,34]. Reliance on AI as a stand-alone diagnostic system without oversight is ethically contentious and may have legal implications. Addressing these concerns requires clinicians to ensure patient safety, acquire ethical AI usage skills, and advocate for robust legal frameworks to guide AI integration [32]. Ultimately, developing unbiased, transparent AI systems with robust human oversight is essential for ensuring ethical implementation and maximizing clinical utility.

Future research should focus on expanding the data set to include a more diverse range of clinical cases. Additionally, integrating multimodal imaging techniques, such as combining PA with cone-beam computed tomography or panoramic radiographs, could provide a more comprehensive diagnostic framework. Exploring the potential of ensemble learning by combining YOLOv8 with advanced segmentation models could further enhance detection and localization performance. This study establishes a strong foundation for the clinical application of YOLOv8 and Mask R-CNN in dental diagnostics. Addressing the outlined limitations and expanding future research will be vital to fully realizing their potential across diverse clinical contexts. Such future research will contribute to the transformative and innovative impact of AI in dentistry.

## 5. Conclusions

This study demonstrates the clinical potential of YOLOv8 and Mask R-CNN in detecting and localizing FEIs and RCTs with high accuracy and reliability. By leveraging the complementary strengths of these models—YOLOv8 for rapid detection and Mask R-CNN for precise segmentation—the research provides a robust framework for integrating advanced AI models into routine dental diagnostics. The inclusion of observer comparisons further demonstrates the practical applicability of these AI models by benchmarking their performance against clinician expertise.

This study serves as a step forward in the integration of AI into dental practice, offering innovative solutions to long-standing diagnostic challenges. Integrating these models into clinical workflows can enhance treatment planning and patient outcomes, particularly for less experienced clinicians. Expanding research in this field will be critical for maximizing the transformative potential of AI in dentistry and ensuring its ethical and effective implementation in diverse clinical settings.

## Figures and Tables

**Table 1:** Comparison of Performance Metrics between YOLO v8 and Mask-R-CNN Models for Detection and Segmentation.

	Accuracy	IoU (Intersection Over Union)	mAP50	Interference Time
YOLO v8	0.973975	1.00	0.989	14.6 ms
Mask-R-CNN	0.982075	0.99	0.950	88.7 ms
*p*	0.571	0.146	0.020	

**Table 2:** Inter- and Intra-group Comparisons of F1 Scores for Detection of FEI, RCT, and BG by Endodontist A, Endodontist B, Mask R-CNN, and YOLOv8.

	Endodontist A	Endodontist B	Mask-R-CNN	YOLOv8	A-B (*p*)	A- Mask-R-CNN (*p*)	A- YOLOv8 (*p*)	B- Mask-R-CNN (*p*)	B- YOLOv8 (*p*)	Mask-R-CNN-YOLOv8 (*p*)
BG F1 Score	N/A	N/A	0.9893	N/A	---	---	---	---	---	---
FEI F1 Score	0.9947	0.9947	0.9890	1.0000	1.000	0.640	0.447	0.640	0.447	0.272
RCT F1 Score	0.9944	0.9944	1.0000	0.9964	1.000	0.447	0.832	0.447	0.832	0.542
BG- FEI (*p*)	---	---	0.971	---						
BG- RCT (*p*)	---	---	0.064	---						
FEI- RCT (*p*)	0.967	0.967	0.126	0.382						

**Table 3:** Comparison of YOLOv8 and Mask R-CNN in Object Detection and Segmentation.

Feature	YOLOv8	Mask R-CNN
Detection Architecture	Single-stage detector that processes the entire image in a single pass.	Two-stage detector that first generates region proposals and then refines detections and segments objects.
Network Architecture	Uses a CNN backbone with a unified prediction head for bounding boxes and class probabilities.	Utilizes a CNN backbone with a Region Proposal Network (RPN), followed by RoI Align and separate branches for classification, box regression, and mask prediction.
Computational Efficiency	Optimized for speed and efficiency, making it suitable for real-time applications.	More computationally demanding due to its two-stage process, leading to higher precision but slower inference.
Detection and Segmentation Output	Outputs bounding boxes and class scores, with instance segmentation added after v8.	Produces bounding boxes, class labels, and masks for pixel-level segmentation.

## Data Availability

The data supporting the findings of this study are available from the corresponding author upon reasonable request. Due to ethical restrictions, access to the raw data sets is limited to ensure the confidentiality of patient information.

## Supplementary Materials

The following supporting information can be downloaded at: https://www.mdpi.com/article/10.3390/diagnostics15060653/s1, Figure S1: YOLO Model Detection Results; Figure S2: Mask R-CNN Model Detection Results.

## Abbreviations

The following abbreviations are used in this manuscript:

FEI	Fractured Endodontic Instrument
RCT	Root Canal Treatment
AI	Artificial Intelligence
CNN	Convolutional Neural Network
MAP	Mean Average Precision
PA	Periapical Radiograph
ICC	Inter-Class Correlation
BG	Background
IOU	Intersection Over Union
RPN	Region Proposal Network
ROI	Region of Interest
FC	Fully Connected
Conv	Convolutional
TP	True Positive
TN	True Negative
FP	False Positive
FN	False Negative
