# Supplementary material for: Detection of Fractured Endodontic Instruments in Periapical Radiographs: A Comparative Study of YOLOv8 and Mask R-CNN
Source: Diagnostics (Basel). 2025 Mar 7;15(6):653. doi: 10.3390/diagnostics15060653 (PMC11940870; doi:10.3390/diagnostics15060653)
Supplement: Supplementary file 1 [file diagnostics-15-00653-s001.zip › diagnostics-3474436-supplementary.pdf]

Supplementary Materials

For the YOLO model, representative TP examples for FEI and RCT illustrated in Figure S1(A) with green bounding boxes, highlight cases where the models accurately detected the respective structures. A high success rate in TP (true positive) values is achieved when the visual distinctions in the images are as clear as possible. For example, the difference in color and shape between a normal root and a root with a RCT is distinct enough for the model to learn. Similarly, in images of FEI, a shape and color that do not appear in a normal tooth image can be observed.

FP examples for FEI and RCT (Figure S1(B) marked with red bounding boxes) illustrate instances where the models misclassified structures due to variations in shape and contrast. In RCT detection, subtle radiopacity differences led to misinterpretations, causing the model to erroneously identify a canal where none existed. Similarly, in FEI detection, an anatomical protrusion resembling the curvature and size of a FEI resulted in misclassification.

FN cases for FEI and RCT (Figure S1 (C) highlighted by blue bounding boxes) highlight instances where the models failed to detect the respective structures despite their presence. As observed in deep-learning-based models, deviations from expected structural characteristics—such as contrast variations, size differences, and alterations in orientation—negatively impacted detection performance, leading to false negatives.

For the Mask R-CNN model, TP cases (Figure S2, indicated by green masks) confirm its ability to accurately delineate canal boundaries and FEIs, occasionally identifying multiple structures within a single radiograph.

FP cases (Figure S2(B) , marked in red masks) reveal incorrect segmentations in regions without FEI or RCT, often near expected structures, suggesting a heightened sensitivity to contrast variations.

FN cases (Figure S2(C), highlighted in blue masks) further highlight the model's limitations in detecting FEI and RCT under varying conditions, reinforcing the challenges posed by structural inconsistencies within radiographic images.

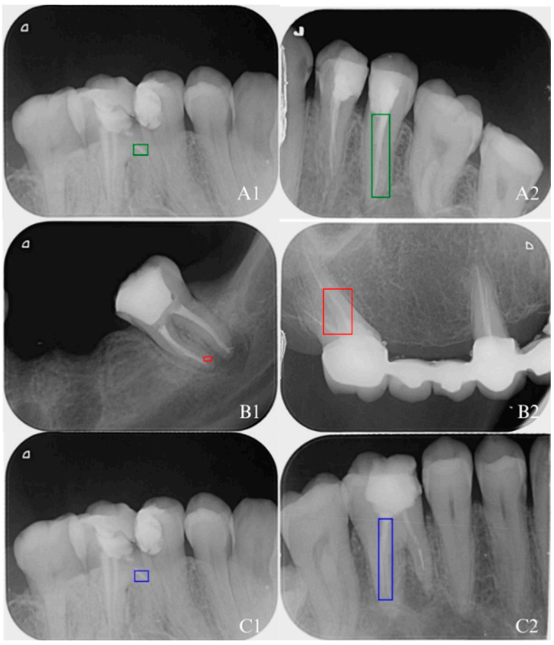

Figure S1: YOLO Model Detection Results. True positive (TP) detections are shown in (A1) for FEI and (A2) for RCT, where the model correctly identified the structures. False positive (FP) cases in (B1) and (B2) demonstrate instances where the model incorrectly marked anatomical features as FEI or RCT, respectively. False negative (FN) cases in (C1) and (C2) highlight situations where the model failed to detect the existing FEI or RCT, despite their presence in the radiographs.

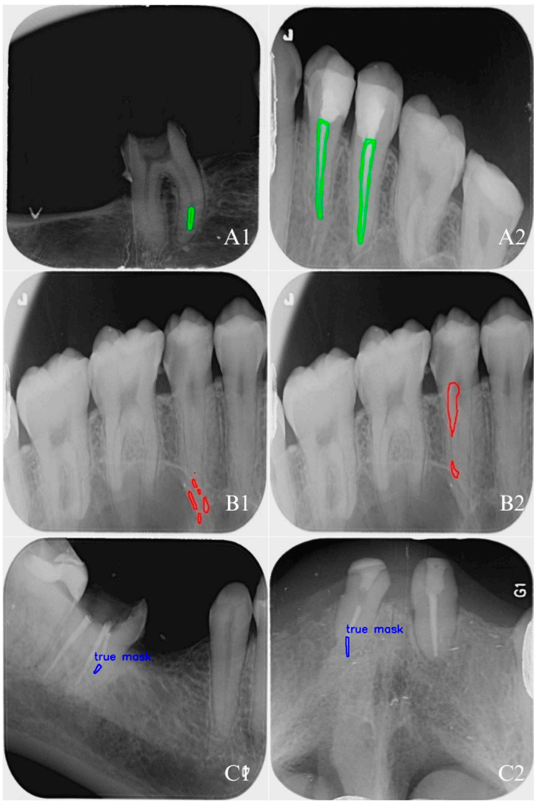

Figure S2: Mask R-CNN Model Detection Results. TP detections in (A1) and (A2) confirm the model's ability to accurately segment FEI and RCT structures. FP cases in (B1) and (B2) show incorrect segmentations where no FEI or RCT was present. FN cases in (C1) and (C2) indicate missed detections, where the model did not recognize the FEI or RCT structures, likely due to variations in contrast, size, or positioning.
